# Supplementary material for: Time-dependent bistability leads to critical slowing down during floral transition in Arabidopsis
Source: Nat Commun. 2026 Aug 25;17:8973. doi: 10.1038/s41467-026-76210-2 (PMC13507123; doi:10.1038/s41467-026-76210-2)
Supplement: Supplementary file 3 [file 41467_2026_76210_MOESM3_ESM.pdf]

nature portfolio

Peer Review File

Time-dependent bistability leads to critical slowing down during floral transition in Arabidopsis

Corresponding Author: Dr Pau Formosa-Jordan

This file contains all reviewer reports in order by version, followed by all author rebuttals in order by version.

Version 0:

Reviewer comments:

Reviewer #1

(Remarks to the Author)
This manuscript explores the concept of bistability critical slowing down as mechanisms underlying biological transitions, applied here to the floral transition in Arabidopsis thaliana. The topic is very timely of great potential interest because dynamic bifurcations critical transitions have been increasingly recognized as important not only across different examples in biology, but more generally for natural systems. In particular, their application to developmental biology can lead to novel conceptual understanding, which can further trigger orthogonal experimental studies leading to new insights. That said, there are specific issues that should be addressed, in my opinion, before the manuscript can be recommended for publication.

1) The core of the manuscript relies on identifying a dynamical transition of a minimal GRN motif that can capture the observed experimental data, the comparison to the experiments seems to be rather qualitative at this stage. This, of course, can be a valid way to test a hypothesis; however, it would be necessary to provide more details for the reader. How have the authors chosen the model parameters? In Figs.2, 4 there are several graphs with a direct comparison between the model the experiments but it is unclear how this has been achieved if it is not clearly stated in the manuscript how the models have been parameterized. Is it a normalization of the model curve that is presented?
2) That said, to quantify the experimental curves of the AP2 profiles in the WT mutant conditions, is it possible to fit i.e. double-exponential or a different function, in order to extract the different that are characteristic for the curve how they change over the mutants, compare maybe the fold changes to those of the model to assess the critical slwoing down the emergence of the second slow time scale in the system?
3) From a biological point of view, what is S? The authors refer to it as an 'age', but what does this mean? Should we assume that the system crosses the bifurcation with cell growth division, or? It would be of great help for the reader if this could be specified or speculated about in terms of its biological meaning. Especially since the authors relate the transition across the bifurcation with a timing mechanism, which, of course, is crucial in development.
4) Can the authors describe/speculate why there are differences between their proposed model the experimental data in Figs. 5D, E G in particular? Is it possible to envision another mechanism in contrast to crossing an HB an SN? Would it be possible that one or multiple SNICs would result in the same temporal profile?

Minor point:
An important reference, in my opinion, that relates to the current work is from Huang's group titled "Time-dependent saddle–node bifurcation: Breaking time the point of no return in a non-autonomous model of critical transitions."

(Remarks on code availability)

Reviewer #3

(Remarks to the Author)
The authors argue, through an approach that combines experimental evidence with bifurcation analysis of simplified deterministic mathematical models, that a time-dependent bistable switch between APETALA2 the floral activators

SUPPRESSOR OF OVEREXPRESSION OF CONSTANS 1 FRUITFULL can account for the dynamics of the floral transition in Arabidopsis. They contend that a slowdown detected in the inhibitor dynamics is consistent with a system crossing a critical point of a bistable switch transiently experiencing a ghost attractor. Their results are aimed at demonstrating that only this time-dependent bistability can generate the range of dynamic behaviors measured across different genotypes, including oscillations in the inhibitor. On the basis of these findings, they postulate that time-dependent bistability constitutes a robust dynamical mechanism for regulating developmental transitions.

It is the opinion of this reviewer that the following points should be taken into account by the authors of the paper:

1. With respect to the final postulate, there exists a substantial body of literature addressing this issue in the context of developmental dynamics in animals. It can therefore be stated that time-dependent bistability indeed belongs to the class of robust dynamical mechanisms that regulate state transitions. In the context of plant developmental dynamics, there are also important results—although not necessarily focused on the floral transition—that explicitly address dynamical analyses incorporating time dependence. It is worth emphasizing that, within the framework for the study of gene regulatory networks proposed by Álvarez-Buylla collaborators, plant developmental transitions can be understood as changes in the dynamical landscape of multistable systems—particularly bistable ones—in which the temporal modulation of regulatory parameters (genetic, hormonal, contextual) confers robustness reproducibility to developmental decisions (Álvarez-Buylla et al., 2008; Álvarez-Buylla et al., 2010; Azpeitia et al., 2014).

References:

Álvarez-Buylla, E. R., Benítez, M., Corvera-Poire, A., Chaos, A., de Folter, S., Gamboa de Buen, A., Garay-Arroyo, A., García-Ponce, B., Jaimes-Miranda, F., Pérez-Ruiz, R. V., Piñeyro-Nelson, A., Sánchez-Corrales, Y. E., & Alvarez-Bañuelos, M. J. (2008). Genetic regulatory network models for plant development. Current Opinion in Plant Biology, 11(1), 1–10.

→ Explicitly introduces the concepts of attractors, multistability, developmental robustness in plants.

Álvarez-Buylla, E. R., Chaos, A., Aldana, M., Benítez, M., Cortes-Poza, Y., Espinosa-Soto, C., Hartasánchez, D. A., Lotto, R. B., Malkin, D., Escalera-Sánchez, E., & García-Ponce, B. (2010). Floral morphogenesis: stochastic explorations of a gene regulatory network state space. PLoS Computational Biology, 6(8), e1000909.

→ Demonstrates that the floral transition corresponds to jumps between attractors in a dynamical landscape, with bistable regimes dependent on context.

Azpeitia, E., Weinstein, N., Benítez, M., Mendoza, L., & Álvarez-Buylla, E. R. (2014). Finding missing interactions of the Arabidopsis thaliana root stem cell niche gene regulatory network. Frontiers in Plant Science, 5, 110.

→ Analyzes the maintenance loss of stability in stem cell niches through nonlinear feedbacks, forming the basis of time-space-dependent bistability.

2. In light of the above, it is recommended that the authors reformulate their statement regarding time-dependent bistability as a mechanism ensuring robustness in developmental transitions, in order to properly acknowledge incorporate the existing body of work.

3. Regarding the choice of Hill dynamics in constructing the formal description of the proposed bistable switch, it is important to explicitly justify the biological meaning of the selected coefficients, beyond merely arguing that they reproduce experimentally observed trends.

4. A gene regulatory network that integrates all flowering transition pathways was proposed in Chávez et al. (2022). This network clearly incorporates the elements included in the model presented in the paper under review. It is therefore necessary for the authors to discuss which considerations are required in order to reduce such a complex network to the simplified model that underpins their argument regarding the presence of time-dependent bistable dynamics.

Reference:

Chávez-Hernández EC, Quiroz S, García-Ponce B Álvarez-Buylla ER (2022) The flowering transition pathways converge into a complex gene regulatory network that underlies the phase changes of the shoot apical meristem in Arabidopsis thaliana. Front. Plant Sci. 13:852047. doi: 10.3389/fpls.2022.852047

5. Although the results presented are interesting, the absence of stochastic noise—both intrinsic extrinsic—in the mathematical description of the biological phenomena under consideration is notable.

Given the dependence of the floral transition on environmental signals, which are inherently subject to noise, it is essential to take this factor into account. It is therefore recommended that the authors incorporate noise into their analysis, at least to demonstrate that the bistable dynamics described in the proposed formal models are robust in the presence of stochastic fluctuations.

(Remarks on code availability)

Version 1:

Reviewer comments:

Reviewer #1

(Remarks to the Author)
The authors have extensively addressed all of my previous concerns comments! In my opinion, the paper brings a fresh perspective to developmental problems in general, I recommend the manuscript for publication.

(Remarks on code availability)

Reviewer #2

(Remarks to the Author)
The study of Rodriguez-Maroto et al aims at understanding of the genetic dynamics underlying the developmental transitions in plants. In particular, it has been found that dynamics of floral inhibitor AP2 activator SOC1 in Arabidopsis during the transition to flowering can be explained by a time-dependent bistable switch. This study relies on experimental data, including spatio-temporal dynamics of AP2 SOC1 expression with associated morphogenetic changes at the shoot apical meristem (SAM), which are well integrated with the modelling reproducing the complex dynamics of these regulators.
In revision which mostly concerned modelling issues, the authors have included additional information on model parameters including biological basis for S parameter additional validations of the bistable models.
Regarding experimental data, the quantification of AP2 SOC1 expression in the WT several mutants is very rigorously conducted with global (tissue-level) local (cell-level) approaches multiple replicates. That provides a strong experimental support for critical slowing down of AP2 expression over floral transition creates a solid basis for the validation of bistable model. Worth noting, insightful analysis of mutants leads to the model extension by AP2 self-repression the spatial aspect including various dynamics of flowering regulators in different SAM regions (central vs peripheral).
Altogether, the manuscript represents a truly interdisciplinary study that enables deeper understanding of genetic mechanisms underlying developmental transitions, as such will be of great interest to both experimental theoretical biologists. I find the manuscript to be of high quality except for a few comments below, I have no additional issues.
Minor comments:
1. Fig. 2A-B. SAM shapes are not always well visible. To capture geometry changes during the floral transition, I would suggest outlining their contours. for clarity, to indicate with arrowheads all visible flower primordia (also those after 19LDs). It also applies to Fig. 3.
2. Line 212. In the sentence: "…leaf primordium formation ceases at the SAM tip…' , please delete 'tip' as it suggests that leaves are formed at the SAM central zone.
3. As it has been noticed that AP2 expression varies between central SAM region the periphery with emerging primordia (lines 324-335), for clarity, it would be worth mentioning earlier in the main text that quantification of AP2 expression (e.g. in Figs 2C 3C, H) concerns the central SAM region only.
4. Supp. Information, line 44 (Figure S1 legend) lines 271-272 (Figure S10 legend): Did the authors really use a membrane marker for cell visualization? In the Methods section (line 906), it is reported that cell walls were stained with Renaissance 2200 dye.

(Remarks on code availability)

Reviewer #3

(Remarks to the Author)
I appreciate the modifications that have been made in response to the comments I raised during the first round of this review process. The manuscript has improved in scientific quality and, consequently, in its usefulness to the community dedicated to the study of the genetic regulation of biological developmental processes.

(Remarks on code availability)

Open Access This Peer Review File is licensed under a Creative Commons Attribution 4.0 International License, which permits use, sharing, adaptation, distribution and reproduction in any medium or format, as long as you give appropriate credit to the original author(s) and the source, provide a link to the Creative Commons license, and indicate if changes were made.
In cases where reviewers are anonymous, credit should be given to 'Anonymous Referee' and the source.
The images or other third party material in this Peer Review File are included in the article's Creative Commons license, unless indicated otherwise in a credit line to the material. If material is not included in the article's Creative Commons license and your intended use is not permitted by statutory regulation or exceeds the permitted use, you will need to obtain permission directly from the copyright holder.
To view a copy of this license, visit https://creativecommons.org/licenses/by/4.0/

Response to Reviewers

We have responded to each of the editor's and reviewers' comments in the text below. The comments that we received appear verbatim in black font and our responses are below each comment in green font. We believe that the changes that we have made to the manuscript to answer the reviewers' comments have greatly improved the text, and we would like to take this opportunity to thank the reviewers for their constructive suggestions.

Editor comment:

Thank you again for submitting your manuscript "Time-dependent bistability leads to critical slowing down during floral transition in Arabidopsis" to Nature Communications. We have now received reports from 2 reviewers and, after careful consideration, we have decided to invite a major revision of the manuscript.

While the reviewers recognize the conceptual novelty of your work and its potential to advance understanding of developmental dynamics in plants, they have raised several important concerns. These include the need for clearer explanation of model parameterization and normalization, quantitative analysis of AP2 profiles to support claims of critical slowing down, and a more explicit biological interpretation of the variable S. Both reviewers emphasize the importance of situating your findings within existing literature on time-dependent bistability and multistability in plant development, as well as justifying modeling choices such as Hill coefficients. Additionally, discussion of how your simplified model relates to comprehensive flowering transition networks, and consideration of stochastic noise to test robustness under realistic conditions, are recommended. Despite these issues, the study is compelling and of interest to the community of developmental timing in general, and we would be happy to consider a revised version, provided that all reviewer concerns are thoroughly addressed. We would like to emphasize that each remaining point is important and expect the revision to respond to them in full.

If you feel that you are able to comprehensively address the reviewers' concerns, please provide a point-by-point response to these comments along with your revision. Please show all changes in the manuscript text file with track changes or colour highlighting. If you are unable to address specific reviewer requests or find any points invalid, please explain why in the point-by-point response.

We were pleased that the editor found the manuscript of interest and invited a revised version. As requested, we have addressed each of the points raised by the reviewers in our following comments, including those specifically highlighted by the editor. We have also addressed the formatting issues mentioned by the editor and have uploaded all of the requested files, including the Summary report and the Code and Software Submission Checklist. To comply with the Nature Communications guidelines, we have also added the new Supplementary Table 14 with the sample size for each experiment and genotype in the Supplementary information file.

REVIEWER COMMENTS

Reviewer #1 (Remarks to the Author):

This manuscript explores the concept of bistability and critical slowing down as mechanisms underlying biological transitions, applied here to the floral transition in Arabidopsis thaliana. The topic is very timely and of great potential interest because dynamic bifurcations and critical transitions have been increasingly recognized as important not only across different examples in biology, but more generally for natural systems. In particular, their application to developmental biology can lead to novel conceptual understanding, which can further trigger orthogonal experimental studies leading to new insights. That said, there are specific issues that should be addressed, in my opinion, before the manuscript can be recommended for publication.

We appreciate the reviewer's interest in the manuscript and their supportive comments regarding the importance of dynamical bifurcations and critical transitions in biology and natural systems. We have addressed each of the specific comments below.

1) The core of the manuscript relies on identifying a dynamical transition of a minimal GRN motif that can capture the observed experimental data, and the comparison to the experiments seems to be rather qualitative at this stage. This, of course, can be a valid way to test a hypothesis; however, it would be necessary to provide more details for the reader. How have the authors chosen the model parameters?

We agree that providing a more quantitative foundation for our parameter selection would help the reader to evaluate the model more thoroughly. To address this, we have significantly expanded the documentation of the model parametrisation of the Toggle-switch model with a negative feedback loop. Specifically:

- **New Model parametrisation subsection**: we have added a dedicated section in the Methods that details the parsimonious and phenomenological logic used. This includes the biological basis for the degradation (d_i) and activation (α_i) rates, as well as for the equilibrium constants (k_{XZ}), ensuring the model respects known biochemical hierarchies (e.g., the coordinated repression of AP2 by both the age signal and the floral activator genes or the stronger contribution of the photoperiodic pathway under inductive light conditions compared to that of the ageing pathway).

- **Quantitative parameter exploration (Figure S22)**: we now include a systematic exploration of (α_0,α_1) and (d_3,k_{FS}) parameter planes (as well as an inspection of optimal time-scaling parameters). We have used the Root Mean Square Error (RMSE) to evaluate the "mathematical optimality" of our sets. As shown in Figure S20, the selected parameter sets are located within the regions of minimum RMSE for each genotype. See *Methods: Model parametrisation* for further details.

- **Biological vs. mathematical optimality**: In our system, we aim to fit parameters for the whole dynamics of the system over time, so the optimisation of the parameter selection is a more complex task than in a system in which a single output (e.g., flowering time or growth) is to be optimised via parameter fitting. We explicitly discuss

the tradeoffs between minimising RMSE and preserving the system's dynamical features (see *Methods: Model parametrisation*). We found that "mathematically optimal" sets with even lower RMSE values often failed to capture key dynamical features measured in experiments on late-flowering mutants, such as the "plateau" (critical slowing down) during the AP2 reduction in *soc1* and *soc1 ful* mutants or reduction in activator strength (α_0 and SOC1p^{ss}) compared to wt. The parameter set selected thus represents an optimal balance that captures both the data points (mathematical fit) and the essential dynamical features that define the floral transition in wt and mutant genotypes.

- **Temporal scaling**: we have clarified the linear temporal rescaling ($t_{exp}= t_{num}\cdot\gamma$+offset) used to align the dimensionless model time with the experimental LDs. By using a shared reference point (AP2-VENUS basal levels at 17LDs in wt), we ensure that delays in floral activator mutants emerge naturally from the system's dynamics rather than individualised curve stretching.

We believe that these additions further strengthen the text and clarify the semi-quantitative framework used to parametrise the model.

In Figs. 2, 4 and 5 there are several graphs with a direct comparison between the model and the experiments but it is unclear how this has been achieved if it is not clearly stated in the manuscript how the models have been parameterized. Is it a normalization of the model curve that is presented?

Regarding the parametrisation, see our answer above. We thank the reviewer for also highlighting the need for clarity in how the model simulations and experimental data were compared in these figures. To address this, we have implemented the following changes:

- **Expanded Methods documentation**: within the new *Model parametrisation* subsection, we have explained how normalisations for simultaneous comparison of model and results were implemented (pages 18 and 19):

"Consistency in normalisation was maintained across all datasets: AP2-VENUS data curves in wt, *ful*, *soc1*, and *soc1 ful* were normalised to the maximum median value reached in wt (which occurs at 7LDs), and AP2 model simulations (dashed lines) were similarly individually normalised to their maximum initial values. This value is identical for wt, *ful*, *soc1*, and *soc1 ful,* and, as in the experiments, also corresponds to the initial value (AP2 at t=0). This approach ensures that the normalised units represent a consistent and comparable relative concentration of the repressor across all genotypes."

Furthermore, the Tissue quantification and Single-cell Quantification subsections have also been updated to describe the normalisation procedures used for the experimental datasets (see Methods section *Fluorescence quantification* and then, *Tissue quantification* and *Single-cell quantification* on page 24).

- **Explicit normalisation details in figure legends:** the legends of Figures 2, 4, and 5 have been updated and now explicitly state information about the normalisation for both AP2-VENUS fluorescence data and the AP2-model repressor variable.

- **Normalisation information in tables**: to provide a direct and accessible reference for the reader, we have added two new Supplementary Tables (Suppl. Tables 3 and 4) containing the details of the normalisation to every experimental data curve and model simulation presented in the manuscript, including Figures 2, 4 and 5.

2) That said, to quantify the experimental curves of the AP2 profiles in the WT and mutant conditions, is it possible to fit i.e. double-exponential or a different function, in order to extract the different that are characteristic for the curve and how they change over the mutants, and compare maybe the fold changes to those of the model to assess the critical slowing down and the emergence of the second slow time scale in the system?

   We thank the reviewer for this insightful suggestion. We agree that a systematic characterisation of the AP2 experimental dynamics is essential to move beyond a qualitative analysis and formally demonstrate the existence of a second, slower timescale associated with critical slowing down in the system. To address this, we have implemented a rigorous curve-fitting and analytical framework to characterise AP2-VENUS dynamics in wt and activator mutants. Our revisions include the following:

   - **Quantitative comparison of fitting models (Figure S7 and Suppl. Tables 5 and 6):** As suggested, we tested multiple functions to describe the AP2 decline, including a single exponential, a sum of two exponentials, and a sum of two arctangent functions. Despite a priori good statistical fits, both single and double exponential models failed to capture the plateau phase in AP2 reduction observed in the data. This failure is objectively demonstrated by the residuals in both experimental models (Figures S7C and S7D), which peak specifically at the transition point after the plateau (14-17LDs in wt; 21LDs in *soc1*).

     In contrast, a sum of two arctangent functions, which naturally accommodates two distinct transition phases separated by a stagnation period, outperforms the exponential model (RMSE = 0.024 in Suppl. Table 5). This parametrisation allows us to unequivocally distinguish the incipient effect of the second timescale in wt and its significant accentuation in *soc1* (Figures S7E).

   - **Characterisation of the critical slowing down through analytical derivatives**: to extract dynamical characterisations from these fits, we computed the analytical derivatives of the sum-of-arctangent functions (Figure S7F). This analysis enabled the quantification of two key features of the ghost-attractor bottleneck: its duration ($\Delta\tau$), defined as the temporal distance between the two maximum rates of decline; and the minimum transition velocity ($|v_{min}|$), which corresponds to the absolute minimum speed of AP2 reduction during the critical slowing down phase. We found that $\Delta\tau$ more than doubles in *soc1* compared to wt (Figure S7G) and a reduction of >50% in $|v_{min}|$ in the floral activator mutant. These metrics provide an objective,

model-independent quantitative signature of the influence of the ghost attractor and the existence of a second timescale within the experimental data.

- **Comparison with theoretical model dynamics (Figure S8)**: We also expanded the theoretical analysis of the influence of the ghost attractor in the system's dynamics by comparing the velocity in numerical simulations with experimental velocities. Hence, we compared the derivatives of model numerical simulations (Figure S8G) with both the derivatives of interpolated AP2-VENUS curves (Figure S8H) and discrete ratios (Δ[AP2]/Δt) from normalised raw measurements (Figure S8I). This comparison reveals that the "stagnation" observed in the activator mutants (especially in *soc1* and *soc1 ful*) is caused by a reduction in the speed of the decline and results in a measurable increase in the time the system is under the influence of the ghost attractor (τ_g) compared to wt (Figures S8K and S8L).

These characterisations are summarised in the new Methods section "*Characterisation of critical slowing down in experimental data and simulated genotypes"* (pages 24 and 25).

We have incorporated these analyses into the Results section (pages 7 and 8) and significantly expanded the characterisation of the ghost attractor (Figures S7, S8, and Suppl. Tables 5 and 6). We believe that these additions clarify that the second timescale associated with the ghost attractor – and by extension, the time-dependent bistability mechanism – is not merely a theoretical prediction of the model, but a quantifiable feature of the experimental AP2-dynamics.

3) From a biological point of view, what is S? The authors refer to it as an 'age', but what does this mean? Should we assume that the system crosses the bifurcation with cell growth and division, or? It would be of great help for the reader if this could be specified or speculated about in terms of its biological meaning. Especially since the authors relate the transition across the bifurcation with a timing mechanism, which, of course, is crucial in development.

We thank the reviewer for highlighting the need for further explanation of the meaning of the age-signal variable, *S*. This variable accounts for the ageing signal mediated by the miR156-SPL module and represents the physiological age of the plant. However, it should not be interpreted as growth or cell division itself, but has been genetically linked to the abundance of miR156, a negative regulator of SPL transcription factors. Biologically, *S* captures the abundance of SPLs, which gradually increase over time at the SAM as a result of the age-dependent decline of miR156, a prerequisite for the floral transition to proceed. In the model, *S* dynamics increase toward saturation, mirroring the mechanism by which SPLs activate *F* (*FUL* and *SOC1*) and *MIR172,* which in turn drive the direct repression of *A* (*AP2*). In the spatial version of the model, the *S* variable also diffuses through space, accounting for the spatial movement of miR172 through plasmodesmata during floral transition. Mathematically, *S* also serves as an external control parameter (*S**) for this developmental transition. Therefore, in this framework, the crossing of the bifurcation as the control parameter *S** increases would represent the accumulation of SPL levels at the SAM at which the plant's internal state shifts from vegetative to reproductive competence. *S* behaves as a proxy for developmental progression during

floral transition, allowing the system to cross the bifurcation as a result of internal physiological changes, rather than simply as a function of external time.

To address this comment of the reviewer and to improve the clarity of the interpretation of this variable, we have updated the Results section to explicitly define *S* in the context of the miR156-SPL module (page 4). Additionally, the discussion of model equations in the Methods section has been expanded to ensure a clearer interpretation of *S* (page 15).

4) Can the authors describe/speculate why there are differences between their proposed model and the experimental data in Figs. 5D, E and G in particular?

We thank the reviewer for the observation regarding the differences between the model's results and certain experimental data points.

Regarding the mismatch observed at 7LDs in the *ful* genotype comparison with the simulations (Figure 5D), this is due to technical constraints during data acquisition at this specific time point. Several samples exhibited image artefacts due to technical issues such as leaf primordia covering the SAM or tissue breakage. Given that the AP2-VENUS concentrations measured in wt, *soc1*, and *soc1 ful* were similar at 7LDs, we believe it is biologically consistent to infer a similar value for *ful*. Under this interpretation, we believe the discrepancy between the model and the experimental data at this time point is therefore not relevant. Detailed information about the processing of 7LDs data samples in *ful* was included within its corresponding code for quantification.

The differences observed in the *soc1* and *rAP2* comparisons (Figures 5E and 5G) stem from the phenomenological nature of the modelling approach used. Our goal was to capture the essential regulatory logic of the floral transition using a parsimonious set of variables. One key simplification was the grouping of SOC1 and FUL into a single activator entity. While these related MADS-box transcription factors share similar roles acting as transcriptional repressors or AP2, they exhibit subtle differences in activation timings. These timing offsets could influence the specific concentration of AP2 at which the system's dynamics begin to slow, as observed in experimental observations (Figure 3C). While a model treating both factors as a single variable may not capture these fine-scale temporal shifts (as seen in Figure 5E), it remains highly effective at capturing the overarching qualitative behaviour of the system (slower floral transition in *soc1*) and key dynamical features in each genotype (accentuated plateau in AP2 reduction in *soc1*).

Similarly, the rAP2 genotype (Figure 5G) represents the strongest perturbation of the GRN analysed in the manuscript. We believe the discrepancy in this genotype arises from the absence of an explicit variable for miR172 and the potential influence of other AP2-LIKE genes, which are known to be able to repress this inhibitor and whose role could be accentuated in contexts with severe deregulation, such as in rAP2 plants. Furthermore, in the *rAP2* mutant, the lack of miR172 regulation could also directly influence AP2-self regulation, adding another layer of complexity to the system.

We have expanded the Discussion section to more explicitly acknowledge these limitations (page 14). We also emphasise that while increasing the model's complexity (e.g., adding another activator variable) could improve quantitative accuracy, it would

likely obscure the study of the dynamical mechanism underlying floral transition that is the focus of this study. As stated in the text (page 14):

“ [...] *the primary goal of this study was not to perfectly fit experimental data, but rather to understand the fundamental nature of the floral transition dynamics and explain the range and likelihood of its possible behaviours*.”

We believe that the changes we have made to the Results and the Discussion specifically address the differences mentioned by the reviewer, but also consider that the robust qualitative agreement displayed across such a wide range of genotypes demonstrates that the biologically grounded simplifications used successfully capture the core mechanism of floral transition.

Is it possible to envision another mechanism in contrast to crossing an HB and an SN? Would it be possible that one or multiple SNICs would result in the same temporal profile?

We appreciate the reviewer’s suggestion to consider a Saddle-Node on Invariant Circle (SNIC) bifurcation. As the reviewer points out, an SNIC bifurcation is another theoretical mechanism through which a temporal profile similar to that of the inhibitor variable (AP2p) could emerge. In contrast to a Hopf bifurcation (HB) in which the amplitude of the oscillations gradually shrinks to zero, in an SNIC, the amplitude remains high (Reference 11 in main text). Additionally, the period experiences a dramatic increase in an SNIC, whereas no changes in frequency are associated with an HB. Considering the well-described features of HB and SNIC bifurcation types, we performed an exhaustive parameter exploration to determine if our system could support an SNIC bifurcation. This analysis proved unsuccessful, with no parameter configuration tested able to generate an SNIC bifurcation, either starting from the current parameter settings or from entirely different regions.

We believe that the inability to find an SNIC bifurcation is due to an inherent characteristic of the specific GRN topology we are modelling, which prevents the SNIC from occurring. In the modelled system, the presence of a limit cycle tends to converge to a supercritical Hopf bifurcation as the primary mechanism to change its stability, before the oscillations can reach the saddle-point required for an SNIC. The age variable, *S*, plays a decisive role in enforcing this behaviour. The initial decrease in AP2p is due to the repressor role of *S* on the inhibitor. In this first stage, the activator variable (SOC1p) not only starts from a zero initial condition, but is also under heavy repression from the inhibitor. Therefore, *S* has an important role in the repression of AP2p at these stages, and leads to a decrease in amplitude in the oscillations of the inhibitor, which is immediately reinforced by the progressive increase in the activator, which is also triggered by S. This specific sequence of events, especially the initial role of S in repressing AP2p, explains why the system is strongly biologically “biased” toward reducing the amplitude of the oscillations in the inhibitor before reaching the saddle node, and hence, crossing a supercritical Hopf bifurcation instead of an SNIC.

This behaviour remains robust across wt and activator mutants (*ful*, *soc1,* and *soc1 ful* double mutant). However, we also investigated whether the rAP2 genotype could provide the conditions necessary for an SNIC in the system (see Figure below). In rAP2, the

resistance of the inhibitor to miR172 is translated as the absence of *S* repression on AP2 dynamics. This absence of age inhibition allows the system to maintain oscillations in the inhibitor without a significant decrease in the amplitude for a longer period, a feature we observed experimentally (Figures 4G–I). We hypothesised that this could potentially allow the limit cycle to collapse either with the saddle node bifurcation itself (SNIC) or with its unstable branch (homoclinic bifurcation). While our simulations in this scenario generated dynamical responses that visually resembled those of an SNIC in terms of sustained amplitude of the oscillations, a closer inspection of the bifurcation diagrams revealed a more complex reality (see Figure below). Because the system is high-dimensional (effectively four-dimensional when *S* is treated as a control parameter, *S**), the "clash" between the oscillations and the unstable solution of the saddle only appeared in certain variables (AP2m and AP2c in the Figure below). Furthermore, even in these cases, no increase in the period of the oscillations was detected in numerical simulations, which is one of the identifying dynamical features of an SNIC or a homoclinic bifurcation. Hence, despite the resemblance, the system also failed to show an SNIC bifurcation for *rAP2*.

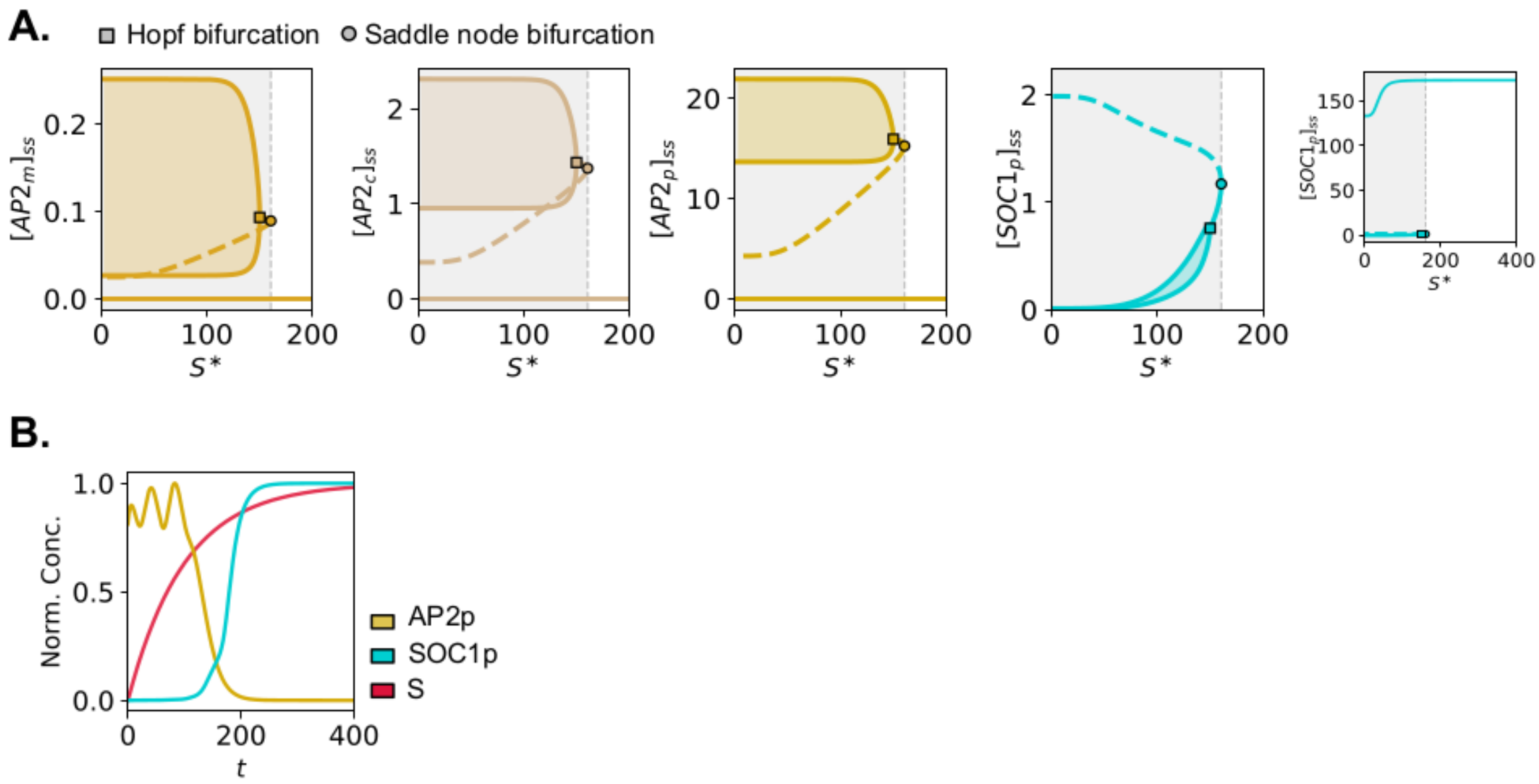

Figure for reviewer. Search for SNIC dynamics in the toggle-switch model with autoinhibition when age-repression on AP2 is deleted. A) Bifurcation diagrams for model variables AP2m, AP2c, AP2p, and SOC1p as a function of the age-associated signal control parameter (*S**) when there is no repression of *S* on AP2 (*rAP2* scenario). Solid lines represent stable steady states, dashed lines represent unstable states. Coloured squares and circles indicate Hopf and saddle-node bifurcation points, respectively. On the right, a zoomed-out bifurcation diagram of SOC1 showing the upper stable branch. The collapse of the limit cycle oscillations and the unstable branch of the saddle-point bifurcation does not happen for the four variables, which denies the presence of an SNIC. B) Numerical simulations showing the temporal dynamics of the activator (SOC1p, cyan), inhibitor (AP2p, yellow), and age variable (*S*, red), individually normalised by their maximum. Although the amplitude of the oscillations does not seem to shrink, which could be interpreted as an SNIC feature, no increase in the period is observed, ruling out the possibility of an SNIC bifurcation. Parameters: $\alpha_1 = 10$, $\alpha_2 = 1.0$, $\alpha_3 = 1.0$, $\alpha_4 = 0.3$, $\alpha_0 = 8.0$, $d_1 = 0.1$, $d_2 = 0.065$, $d_3 = 0.09$, $d_1 = 0.06$, $\beta_S = 3.0$, $d_S = 0.01$, $k = 8.0$, $k_{AF} = 0.25$, $k_{FA} = 1.5$, $k_{FS} = 38.0$, $n = 10.0$, and $n_2 = 4.0$.

This bifurcation analysis was useful to understand the constraints of the system. The difficulty in finding the SNIC or a homoclinic bifurcation appears to be driven primarily in

this context by the activator variable, SOC1p, rather than the inhibitor (AP2p). The activator (initial) basal levels are naturally too low to be able to generate high amplitude oscillations that are required to "hit" the saddle-node bifurcation point or the unstable intermediate branch without first completely shrinking through the Hopf bifurcation. We tested extreme measures to bypass this, such as drastically increased Hill coefficients (n, $n_2 > 10$) to ensure "switch-like" nonlinearities that would force the limit cycle to persist near the saddle-node bifurcation point, or a very strong inhibitor expression rate (α_1) to counteract the effects of *S* and SOC1p repression. However, the system always ended up reaching the Hopf bifurcation first. None of these adjustments successfully translated into a higher amplitude in the activator. Attempts to increase the amplitude of the oscillations in the activator by increasing the activator's expression rate (α_0 or α_4) were also ineffective and did not lead to an SNIC bifurcation.

This inability of the system to cross an SNIC bifurcation is also supported by the experimental observations. The lack of measured fluctuations in SOC1 protein dynamics in vivo argues against an SNIC and in favour of a Hopf bifurcation. For an SNIC to happen, activator levels should oscillate - and keep the amplitude high - until they physically collide with the unstable-steady branch of the saddle. This lack of fluctuations in SOC1 thus would support a model in which the system passes through a supercritical Hopf bifurcation. It is precisely the nature of the interactions in the GRN, the age signal triggering the repression of the inhibitor first and then, promoting the expression of the floral activators, that inherently favours the emergence of a Hopf bifurcation over an SNIC. In the future, new reporter lines will enable a more detailed analysis of the protein activator dynamics (SOC1 and FUL) in the different genotypes, which should contribute to further clarifying this dilemma between Hopf and SNIC.

Minor point:
An important reference, in my opinion, that relates to the current work is from Huang's group titled "Time-dependent saddle–node bifurcation: Breaking time and the point of no return in a non-autonomous model of critical transitions."

Thanks for this. The reviewer is right that we should have cited this manuscript from the Huang group, and we apologise for that oversight. We have now included this article in the bibliography (Reference 17).

Reviewer #3 (Remarks to the Author):

The authors argue, through an approach that combines experimental evidence with bifurcation analysis of simplified deterministic mathematical models, that a time-dependent bistable switch between APETALA2 and the floral activators SUPPRESSOR OF OVEREXPRESSION OF CONSTANS 1 and FRUITFULL can account for the dynamics of the floral transition in Arabidopsis. They contend that a slowdown detected in the inhibitor dynamics is consistent with a system crossing a critical point of a bistable switch and transiently experiencing a ghost attractor. Their results are aimed at demonstrating that only this time-dependent bistability can generate the range of dynamic behaviors measured across different genotypes, including

oscillations in the inhibitor. On the basis of these findings, they postulate that time-dependent bistability constitutes a robust dynamical mechanism for regulating developmental transitions.

It is the opinion of this reviewer that the following points should be taken into account by the authors of the paper:

1. With respect to the final postulate, there exists a substantial body of literature addressing this issue in the context of developmental dynamics in animals. It can therefore be stated that time-dependent bistability indeed belongs to the class of robust dynamical mechanisms that regulate state transitions. In the context of plant developmental dynamics, there are also important results—although not necessarily focused on the floral transition—that explicitly address dynamical analyses incorporating time dependence. It is worth emphasizing that, within the framework for the study of gene regulatory networks proposed by Álvarez-Buylla and collaborators, plant developmental transitions can be understood as changes in the dynamical landscape of multistable systems—particularly bistable ones—in which the temporal modulation of regulatory parameters (genetic, hormonal, and contextual) confers robustness and reproducibility to developmental decisions (Álvarez-Buylla et al., 2008; Álvarez-Buylla et al., 2010; Azpeitia et al., 2014).

   References:

   Álvarez-Buylla, E. R., Benítez, M., Corvera-Poire, A., Chaos, A., de Folter, S., Gamboa de Buen, A., Garay-Arroyo, A., García-Ponce, B., Jaimes-Miranda, F., Pérez-Ruiz, R. V., Piñeyro-Nelson, A., Sánchez-Corrales, Y. E., & Alvarez-Bañuelos, M. J. (2008). Genetic regulatory network models for plant development. Current Opinion in Plant Biology, 11(1), 1–10.
   → Explicitly introduces the concepts of attractors, multistability, and developmental robustness in plants.

   Álvarez-Buylla, E. R., Chaos, A., Aldana, M., Benítez, M., Cortes-Poza, Y., Espinosa-Soto, C., Hartasánchez, D. A., Lotto, R. B., Malkin, D., Escalera-Sánchez, E., & García-Ponce, B. (2010). Floral morphogenesis: stochastic explorations of a gene regulatory network state space. PLoS Computational Biology, 6(8), e1000909.

   → Demonstrates that the floral transition corresponds to jumps between attractors in a dynamical landscape, with bistable regimes dependent on context.

   Below in our reply, we will refer to the paper as (Álvarez-Buylla et al, 2008b), given that it is from 2008 and not from 2010.

   Azpeitia, E., Weinstein, N., Benítez, M., Mendoza, L., & Álvarez-Buylla, E. R. (2014). Finding missing interactions of the Arabidopsis thaliana root stem cell niche gene regulatory network. Frontiers in Plant Science, 5, 110.

→ Analyzes the maintenance and loss of stability in stem cell niches through nonlinear feedbacks, forming the basis of time- and space-dependent bistability.

We thank the reviewer for pointing out previous reviews and research highlighting time-dependent dynamical analyses in plant development. The original manuscript included references in relation to these topics, e.g. the work by Davila-Valderrain et al. (2015 and 2016) regarding epigenetic landscapes and flowering transitions, or the work from Abley et al (2021) regarding time-bistability on seed germination (see below the full reference names). But we agree it is important to provide more historical and theoretical foundation for those topics whenever possible. The work by Álvarez-Buylla et al. (2008b) regarding stochastic explorations of floral morphogenesis state space provides an excellent new reference that we incorporated into our manuscript (Reference 30).

References mentioned above, which were already included in the original manuscript:

Abley, K. *et al.* An ABA-GA bistable switch can account for natural variation in the variability of Arabidopsis seed germination time. *eLife* **10**, e59485 (2021).

Davila-Velderrain, J., Martinez-Garcia, J. C. & Alvarez-Buylla, E. R. Modeling the epigenetic attractors landscape: Toward a post-genomic mechanistic understanding of development. *Front. Genet.* **6**, (2015).

Davila-Velderrain, J., Martinez-Garcia, J. C. & Alvarez-Buylla, E. R. Dynamic network modelling to understand flowering transition and floral patterning. *J. Exp. Bot.* **67**, 2565–2572 (2016).

2. In light of the above, it is recommended that the authors reformulate their statement regarding time-dependent bistability as a mechanism ensuring robustness in developmental transitions, in order to properly acknowledge and incorporate the existing body of work.

   We thank the reviewer for these thoughtful comments and for highlighting the importance of placing our work within the broader context of dynamical systems in plant development. We agree that the concept of “dynamical landscapes”, where transitions can be understood as shifts between attractors, is a fundamental framework for understanding developmental robustness. We have also updated the Discussion to more explicitly acknowledge how our findings regarding time-dependent bistability and critical slowing dynamics align with the broader framework of multistable systems and parameter-modulated robustness in plant development (pages 12 and 13), which cites Álvarez-Buylla et al. (2008b). We believe these additions strengthen the interdisciplinary context of our study.

   Also, we have reformulated our statement on robustness in the abstract, moving it from the last general statement to the previous sentence. This change reduces the emphasis on robustness in relation to our results in the abstract. Now, the last sentence in the abstract provides instead a summary sentence of our work, as required by the Nature Communications guidelines.

3. Regarding the choice of Hill dynamics in constructing the formal description of the proposed bistable switch, it is important to explicitly justify the biological meaning of the selected coefficients, beyond merely arguing that they reproduce experimentally observed trends.

   We thank the reviewer for the suggestion to further clarify the biological justification for our selected Hill coefficients. In the new Methods section, *Model parametrisation*, pages 18 and 19, we have clarified this parametrisation aspect.

   In our model, the Hill coefficients represent the effective cooperativity and the non-linear "switch-like" nature of the regulatory interactions within the GRN. It has been mathematically demonstrated that in a three-component autoinhibition network, high non-linearity (with effective Hill coefficient $n > 8$) is a structural requirement to generate sustained limit-cycle oscillations (Reference 83). While a single transcription factor binding event rarely exhibits such high cooperativity, in a phenomenological model, this value accounts for the integrated effect of multiple regulatory layers (e.g., chromatin remodelling, protein multimerisation, etc.), which together can sharpen the developmental response.

   For the remaining interactions, we selected a Hill coefficient of $n = 2$. This value represents a standard assumption of moderate cooperativity, allowing the model to capture essential non-linearities without over-parametrising the system or assuming extreme sensitivity where it is not biologically evidenced.

4. A gene regulatory network that integrates all flowering transition pathways was proposed in Chávez et al. (2022). This network clearly incorporates the elements included in the model presented in the paper under review. It is therefore necessary for the authors to discuss which considerations are required in order to reduce such a complex network to the simplified model that underpins their argument regarding the presence of time-dependent bistable dynamics.

   Reference:

   Chávez-Hernández EC, Quiroz S, García-Ponce B and Álvarez-Buylla ER (2022) The flowering transition pathways converge into a complex gene regulatory network that underlies the phase changes of the shoot apical meristem in Arabidopsis thaliana. Front. Plant Sci. 13:852047. doi: 10.3389/fpls.2022.852047

   We thank the reviewer for this comment, which highlights the need for further clarification of the rationale behind the formulation of our simplified model. The decision to prioritise a parsimonious model for this complex GRN is based on both biological and theoretical considerations.

   While comprehensive GRN reconstructions, such as those proposed by Chávez et al. (2022), successfully catalogue the extensive set of components involved in the floral transition, they may include functional redundancies. For example, the linear CO-FT

module can be functionally condensed for a SAM-specific model, as FT acts as the primary systemic output of CO. Furthermore, global networks typically include components expressed in distant tissues (both CO and FT are expressed in leaves). Our approach specifically uses the current information on flowering pathways to reduce the system to the effectors in the meristem of the major flowering pathways (*SOC1* and *FUL* as outputs of the *FT* pathway; *AP2* repression and *FUL* activation as major outputs of the ageing pathway). This has the advantage of focusing on a single tissue where floral transition occurs, and from which we have acquired data. It is also based on direct interactions, which reduces the complexity: AP2 binds to the promoters of *SOC1* and *FUL*, and SOC1 and FUL bind to the promoter of *AP2*. The importance of these proteins and genes and their expression patterns is fully supported by genetics and detailed temporal confocal microscopy, as presented here. Focusing on these interactions allows us to obtain detailed data on the temporal accumulation of the proteins across the developmental transition in a single tissue and relate the data to the model.

In terms of a dynamical systems theory perspective, our approach is also fundamentally different in both scope and intent from large-scale reconstructions. While Boolean models are powerful for identifying steady-state configurations in large-scale networks, they can be more limited in their ability to describe the temporal continuity and non-linear thresholds that define developmental “tipping/bifurcation points”.

To investigate the presence of time-dependent bistable dynamics, we focused on a core regulatory motif—a reduced-order ODE model. This simplification is a necessary step to analyse the system as a dynamical switch. By condensing the network into its essential feedback loops, we can apply bifurcation theory to explain key features experimentally observed, such as the 'ghost' attractor dynamics or the precise timing of the AP2 decline, features that are often lost in high-dimensional discrete models.

To address the reviewer’s comment, we have expanded the explanation of the model formulation in the Methods, in the Model equations section (page 15) to clarify these points. We also thank the reviewer for the suggested reference, which we have now incorporated into the revised manuscript (Reference 35) to provide a broader context for flowering GRNs.

5. Although the results presented are interesting, the absence of stochastic noise—both intrinsic and extrinsic—in the mathematical description of the biological phenomena under consideration is notable.

   Given the dependence of the floral transition on environmental signals, which are inherently subject to noise, it is essential to take this factor into account. It is therefore recommended that the authors incorporate noise into their analysis, at least to demonstrate that the bistable dynamics described in the proposed formal models are robust in the presence of stochastic fluctuations.

   We thank the reviewer for this important suggestion. We agree that investigating the role of stochasticity - both intrinsic and extrinsic - is essential for evaluating the robustness of developmental transitions and the dynamical mechanism here proposed.

To address this comment, we have performed comprehensive stochastic simulations across all genotypes (wt, *ful*, *soc1*, *soc1 ful*, *rAP2*, and *ap2*) to analyse the effects of intrinsic and extrinsic multiplicative noise (see new Figures S15 and S16 for intrinsic noise simulations and Figures S17 and S18 for extrinsic stochastic simulations). Three different noise intensities were tested. The temporal dynamics of model variables consistently recapitulated the behaviour observed in the deterministic model, confirming that the bistable switch (with AP2-autoinhibition) mechanism is robust to molecular and environmental perturbations. Furthermore, the increase in noise strength led to increased variability across all genotypes, as expected. Additionally, our analysis near the saddle-node bifurcation revealed that increased noise can destabilise the ghost attractor and lead to earlier vegetative to inflorescence transitions (Figure S19). We have also measured the Quartile Coefficient of Variation (QCV) to illustrate how stochasticity modulates these transient dynamics. These findings have been incorporated into the Results section *AP2 dynamics during floral transition in different genotypes can be explained by the extended toggle-switch model* (page 11).

We believe this addition successfully demonstrates the robustness of our proposed mechanism. Although the interplay between noise and developmental timing is a compelling area for future research, we have kept this analysis focused on validating the deterministic framework to maintain the manuscript's primary focus on the underlying dynamical mechanism.

To acknowledge related literature on stochasticity at criticality, we added the following references:

Fucho-Rius, M. *et al.* Local nearby bifurcations lead to synergies in critical slowing down: The case of mushroom bifurcations. *Phys. Rev. E* **111**, 024213 (2025).

Mojtahedi, M. *et al.* Cell Fate Decision as High-Dimensional Critical State Transition. *PLOS Biol.* **14**, e2000640 (2016).

Response to Reviewers

We have responded to each of the editor's and reviewers' comments in the text below. The comments that we received appear verbatim in black font, and our responses are below each comment in green font. We believe that the adjustments made to the manuscript in response to reviewers' comments have further improved the clarity of the work. We would like to take this opportunity to thank the reviewers for their constructive feedback.

Editor comment:

Apologies for the delay in reaching an editorial decision with your manuscript, and thanks again for submitting your revised manuscript "Time-dependent bistability leads to critical slowing down during floral transition in Arabidopsis" to Nature Communications. We have now received reports from the reviewers who evaluated the original version and a new Reviewer #2. [...], we have decided to invite an additional minor revision of your work.

We appreciate the editor's effort to secure an additional reviewer to evaluate the experimental part. We are pleased that the new reviewer found our work interesting and raised only minor points for revision. As requested, we have addressed each of these comments in the main text and supplementary information, and we reply to the reviewer's comments below.

REVIEWER COMMENTS

Reviewer #1 (Remarks to the Author):

The authors have extensively addressed all of my previous concerns and comments! In my opinion, the paper brings a fresh perspective to developmental problems in general, and I recommend the manuscript for publication.

We appreciate the reviewer's interest in our work and comments on the strength of the manuscript. We are glad the reviewer recommends the manuscript for publication.

Reviewer #2 (Remarks to the Author):

The study of Rodriguez-Maroto et al aims at understanding of the genetic dynamics underlying the developmental transitions in plants. In particular, it has been found that dynamics of floral inhibitor AP2 and activator SOC1 in Arabidopsis during the transition to flowering can be explained by a time-dependent bistable switch. This study relies on experimental data, including spatio-temporal dynamics of AP2 and SOC1 expression with associated morphogenetic changes at the shoot apical meristem (SAM), which are well integrated with the modelling reproducing the complex dynamics of these regulators.
In revision which mostly concerned modelling issues, the authors have included additional information on model parameters including biological basis for S parameter and additional validations of the bistable models. Regarding experimental data, the quantification of AP2 and SOC1 expression in the WT and several mutants is very rigorously conducted with global (tissue-level) and local (cell-level) approaches and multiple replicates. That provides a strong

experimental support for critical slowing down of AP2 expression over floral transition and creates a solid basis for the validation of bistable model. Worth noting, insightful analysis of mutants leads to the model extension by AP2 self-repression and the spatial aspect including various dynamics of flowering regulators in different SAM regions (central vs peripheral). Altogether, the manuscript represents a truly interdisciplinary study that enables deeper understanding of genetic mechanisms underlying developmental transitions, and as such will be of great interest to both experimental and theoretical biologists. I find the manuscript to be of high quality and except for a few comments below, and I have no additional issues.

We appreciate the reviewer's interest in the manuscript and their supportive comments regarding the rigour of the experimental analyses performed and the resulting biological insights. We were pleased that the reviewer considers this to be a high-quality, truly interdisciplinary study that can be of interest to both experimental and theoretical biologists. We have addressed each of their specific comments below:

1. Fig. 2A-B. SAM shapes are not always well visible. To capture geometry changes during the floral transition, I would suggest outlining their contours. And for clarity, to indicate with arrowheads all visible flower primordia (also those after 19LDs). It also applies to Fig. 3.

We thank the reviewer for highlighting the need to improve the visualisation of SAM confocal images during the floral transition. We agree that the outline of the meristem contours can provide relevant information about how SAM morphology evolves during the transition. Furthermore, the outline of the shape of the meristem is critical at timepoints in which the fluorescence reporter signal of the studied transcription factors (SOC1 and AP2) is very low.

Accordingly, we have modified the figures and added a dashed outline indicating the contours of the SAMs in Figures 2A and 2B, 3A, 3B, 3F, 3G, and S10A. Following the reviewer's suggestion, we have also indicated with arrowheads all visible floral primordia in those figures. We have explained the dashed outlines and arrowheads in the corresponding legends.

2. Line 212. In the sentence: "…leaf primordium formation ceases at the SAM tip…' , please delete 'tip' as it suggests that leaves are formed at the SAM central zone.

We thank the reviewer for highlighting this erroneous description in the text. We have corrected the phrasing and deleted "tip". The text now reads "*leaf primordium formation ceases at the flanks of the SAM*" (line 212).

3. As it has been noticed that AP2 expression varies between central SAM region and the periphery with emerging primordia (lines 324-335), for clarity, it would be worth mentioning earlier in the main text that quantification of AP2 expression (e.g. in Figs 2C and 3C, H) concerns the central SAM region only.

We thank the reviewer for the suggestion to explicitly clarify the spatial expression domains targeted by the fluorescence quantification pipeline. To ensure clarity from the beginning, we have added an explicit mention of this the first time the quantification pipeline is introduced in the main text: "*by defining characteristic expression domains within the central region of the SAM (see Methods and Figure S1)*" (line 148). Furthermore, we have updated the figure captions for Figures 2 and 3 to include "*within the SAM central region*", and we have

incorporated the word “central” into the technical description of the pipeline in the Methods section (line 937).

4. Supp. Information, line 44 (Figure S1 legend) and lines 271-272 (Figure S10 legend): Did the authors really use a membrane marker for cell visualization? In the Methods section (line 906), it is reported that cell walls were stained with Renaissance 2200 dye.

The reviewer is right. We apologise for the error and thank the reviewer for the comment. No membrane marker reporter was used in any of the experiments. All meristems imaged were stained with the Renaissance 2200 dye. We have corrected this error in the legends of Figures S1 and S10.

Reviewer #3 (Remarks to the Author):

I appreciate the modifications that have been made in response to the comments I raised during the first round of this review process. The manuscript has improved in scientific quality and, consequently, in its usefulness to the community dedicated to the study of the genetic regulation of biological developmental processes.

We are glad that the reviewer appreciates the modifications made in the revision of the manuscript and values the manuscript's usefulness to the scientific community.
